# Causal role of infectious agents in cancer: An overview

**DOI:** 10.22088/cjim.8.3.153

**Published:** 2017

**Authors:** Jila Masrour-Roudsari, Soheil Ebrahimpour

**Affiliations:** 1 Infectious Diseases and Tropical Medicine Research Center, Health Research Institute, Babol University of Medical Sciences, Babol, Iran.

**Keywords:** Infection, Cancer, Prevention, Virus, Bacteria

## Abstract

Cancer is a complex group of diseases with multiple eventual causes. The underlying causes are not fully known. Thus, learning more about the known causes of cancer is an important issue. Moreover, among these factors, infection and its association to cancers is controversial. Although, it seems that the genome instability of the cells can initiate cancer development. The purpose of this review was to present the role of infection in the development of cancer. Infectious agents, such as hepatitis B (HBV) and C viruses (HCV), Epstein-Barr virus (EBV), human papillomavirus (HPV), human immunodeficiency virus type 1 (HIV-1), Helicobacter pylori (H. pylori) and Streptococcus bovis (S. bovis) contribute to the pathogenesis of different cancers. These cancers include hepatocellular carcinoma, Burkitt's lymphoma, nasopharyngeal carcinoma, cervical cancers, non-Hodgkin lymphoma, Kaposi sarcoma, adenocarcinoma and lymphoma. Screenings of infectious diseases in cancer patients may open up areas of research in the identification of optimizing cancer control strategies.

## Introduction

At the beginning of the last century, it has been known that infectious agents play an essential role in some cancers in animals (1, 2). Recently, infections with specific type of viruses and bacteria have been distinguished as risk factors of developing cancer in humans. According to the International Agency for Research on Cancer (IARC), some of these agents contain hepatitis B(HBV )and C viruses(HCV), Epstein-Barr virus (EBV), human papillomavirus (HPV), human immunodeficiency virus type 1 (HIV-1), Helicobacter pylori (H. pylori) and Streptococcus bovis (S.bovis) (3, 4). Generally, infections are linked to 10% types of cancer (5-7). 

The prevalence of these items is lower in developed countries and higher in developing regions of the world(8). Infectious agents can raise the risk of human cancer with different mechanisms. Agents such as HPV, HBV and EBV acting as direct carcinogens via the expression of oncogenes can inhibit apoptosis and increase cell immortalization (9). In other words, these agents are effective on genome instability of cells and can cause the cell to grow out of control. H. pylori and HCV can cause long-term inflammation in the body (10, 11). This can lead to changes in the affected immune cells and produce inflammatory mediators, which can finally cause cancers.

 Immunosuppression due to HIV-1 infection can downregulate immune response which normally helps protect the body from certain cancers (table 1). The aim of this review is to present the role of infections on cancer and learn more about the association between these agents and cancers.

**Table1.** Infectious Agents, Cancer Sites and Mechanisms of Carcinogenesis

**Reference**	**Mechanisms of Carcinogenesis**	**Cancer Sites**	**Infectious Agents**
(17,19)	Chronic Direct and carcinogens inflammation	Hepatocellular carcinoma	HBV
(26)	Chronic inflammation	Hepatocellular carcinoma	HCV
(34,36)	Direct carcinogens	Burkitt's lymphoma, Nasopharyngeal carcinoma and Hodgkin lymphoma	EBV
(43)	Direct carcinogens	Carcinoma of cervix, vulva anus, oral cavity	HPV
(49)	Immune suppression	Kaposi sarcoma, non-Hodgkin lymphoma, carcinoma of the cervix	HIV
(52,56)	Chronic inflammation	Adenocarcinoma and lymphoma	H.pylori
(58)	Chronic inflammation	Colorectal cancer	S. bovis

**Hepatitis B virus: **Hepatitis B is a virus that spreads by direct contact with infected blood or body fluids, while sharing needles, during unprotected sexual contact, or childbirth (12, 13). According to HBsAg seropositivity and the proportion of population related to this item, the following results were obtained. Approximately 45% of HBV-infected persons live in highly endemic areas (Southeast Asia, China, and Sub-Saharan), 43% reside in intermediate endemic regions (some regions of the Mediterranean, the Middle East, Central Asia, Eastern Europe and South America), whereas, the remaining 12% live in low endemic areas (Australia, Japan, North America and Western Europe) (14). 

Hepatocellular carcinoma (HCC) is associated with cirrhosis related to chronic HBV. *Liver cancer is the tenth most common cancer and the fifth leading cause of cancer death among men (*15*, *16*). It is also the 7th most common cause of cancer death among women.* Hepatocellular carcinoma (HCC) is the most common type of liver cancer, in which 80% cases of this cancer are associated with chronic HBV or HCV infections (17, 18). HBV infection causes HCC development through various mechanisms. HBV-DNA integration into the host genome occurs at clonal tumor expansion and contributes to genomic instability of multiple cancer type-dependent genes. Prolonged expression of the viral regulatory protein HBx disturbs the setting of cellular transcription and proliferation and sensitizes liver cells to carcinogenic factors (19).

**Hepatitis C Virus: **Hepatitis C is transmitted by percutaneous or permucosal exposure to infectious blood or blood-derived fluids of HCV -infected person (20-22). Also, in many cases needle sharing in the process of injection drugs become infected with this agent. Spontaneous HCV clearance happens in up to 10% of infected cases after about six months of infection without treatment. The remaining 80% of persons will develop chronic infection (23). Around 30% of worldwide chronic hepatitis patients will develop cirrhosis of the liver within 20 years. Furthermore, 25% HCC cases are attributable to HCV (24). Worldwide, epidemiological studies showed that the total global HCV prevalence is estimated at 2% (about 170 million people infected with HCV) and Central Africa and Asia are estimated to have high prevalence. The Middle East, East and Southeast Asia, West Africa, Australasia and Eastern Europe with moderate prevalence, while Southern Africa, North America and Western Europe had low prevalence (25). Many research studies have shown that HCV core proteins are oncogenic and their expression in the cell can be engaged in the development of HCC, particularly in immune response deficiency. In the other words, different HCV proteins can interact with host cell proteins in proliferation and differentiation. Numerous documents have shown that replication of HCV replicon RNAs is clearly dependent on cell proliferation. Hepatocytes usually have low proliferation rate and it seems these phenomena leads to HCC (26). Moreover, in patients with chronic HCV, long- term inflammation from host immune response to infected hepatocytes is associated with cirrhosis in which an abnormal liver condition. Cirrhosis is a great risk factor for HCC (27, 28).

**Epstein-Barr Virus: **EBV can be transmitted from person to person by coughing sneezing or by sharing drinking or eating utensils, especially in children (29, 30). In adolescents primary EBV infection often presents as infectious mononucleosis (31, 32). Latent EBV infection is linked to various malignancies in human. EBV is associated with 20% of Burkitt lymphoma in the developed countries and almost all African Burkitt lymphoma, within 50% of Hodgkin lymphoma, 10% stomach carcinomas, almost all endemic nasopharyngeal carcinoma (33). Burkitt's lymphoma occurs endemically in many parts of Africa. Studies show that EBV is involved in the pathogenesis of this lymphoma and the reason for this claim is that all patients with Burkitt's lymphoma have antibodies to EBV antigens and multiple copies of EBV genome can be presented in the monoclonal tumor cells of Burkitt's lymphoma (34). 

Nasopharyngeal carcinoma (NPC) is the most common cancer originating in the nasopharynx. This tumor is rare in most other areas of the world, but the most pockets of high incidence occur in Malaysia, North of Africa and Iceland. The important evidence shows that EBV is engaged in the pathogenesis of nasopharyngeal carcinoma. Furthermore, these issues administrate that multiple copies of the EBV genome can be detected in the malignant cells of this carcinoma (35). EBV can contribute to the development of cancers through several mechanisms. Its attendance in some stages of B-cell development and also its ability to infect epithelial cells, can have pathological outcomes and chances of developing lymphoma and carcinoma (36).

**Human papillomavirus: **HPV is most common sexually transmitted infection via a direct skin-to-skin contact from vaginal, and or oral sex (37-39). Human papillomaviruses are small and double-stranded DNA viruses that infect basal epithelial cells. The 13 HPV types considered to be high-oncogenic risk are; 16, 18, 31, 33, 35, 39, 45, 51, 52, 56, 58, 59 and 66 that can lead to cervical cancer including neck and head cancers (40). 

Head and neck cancer is a group of cancers that starts within the mouth, nose, throat or any parts of salivary gland. Infections with other genotypes known as “low risk” can cause benign or genital warts, which are considered growths on the cervix, vulva and anus in women and the penis, scrotum in men. This means that benign lesions are often caused by low-risk HPV genotypes with genotypes 6 and 11 contributing to more than 90% of genital warts (41). Studies showed that there were almost half a million new cases of cervical cancer and this disease was associated with about 200000 deaths annually in developing countries. The higher prevalence of cervical cancer in these regions may be attributed to the limited or lack of access of women to health care screening programs (42). 

Expression of high-risk HPV E6 and E7 genes is important for the induction of premalignant stages, and leads to malignant progression by destroying genomic constancy. For example, expression of the HPV E6 and E7oncogens can immortalize in primary human keratinocytes considerably (43). 

**Human immunodeficiency virus type 1: **HIV-1 is transmitted by unprotected sex with HIV-infected cases, percutaneous inoculation, prenatal and perinatal exposure of infants from infected mothers and transfusion of infected blood products from HIV-infected individuals (44-46). In 2015, 2.1 million individuals worldwide became newly infected with *HIV (*47*).* The primarily concentrated areas of Sub-Saharan Africa have the highest global burden of infection. The global prevalence of HIV has increased from 31 million in 2002 to 35 million in 2012, while the global incidence has decreased from 3 million in 2002 to 2 million in 2012. Worldwide AIDS-related deaths peaked at 2·3 million in 2005, and decreased to within 1.5 million by 2012 (48). 

HIV infects white blood cell (WBC) known as helper T-cells (Th) and many other immune cells, which weakens immune responses and diminishes the body's ability against infection agents that may lead to cancers. Infected cases have the higher risk of several types of cancer in which three of these cancers such as non-Hodgkin lymphoma, Kaposi sarcoma and cervical cancer were recognized (49). Moreover, some researchers reported the high incidence rate of colorectal cancers in AIDS yet, this cancer does not have a known viral etiology. Kaposi sarcoma as the most common malignancy associated with infection can be presented at different stages of the disease; however it usually occurs when severe immune suppression takes place. HIV-1 infected patients develop non-Hodgkin lymphomas at high frequency (3%) while cervical cancer is considered as one of the most common cause of cancer with more infectious complications in the developing countries (50).

**Helicobacter pylori: **Helicobacter pylori is a gastric pathogen that colonizes about 50% of the world's population which is the primary cause gastritis and peptic ulcer, gastric adenocarcinoma and lymphoma (51, 52). In developed regions, around 60% of adults are infected, yet, the prevalence of infection seems to be decreasing. Besides, in the developing countries the prevalence is higher than 80% (53-55). These bacteria are generally, transmitted person-to-person by saliva. As well as it can be spread by fecal contamination of water. In the developing countries, there is a higher prevalence of infection due to the contaminated water and poor hygiene. H. pylori infection mounts a chronic inflammatory response over several decades area may result in mitotic errors. Virulence factors like cytotoxin-associated gene A (CagA) and vacuolating cytotoxin A (VacA) have been shown to be associated with precancerous gastric lesions and progression to a malignant phenotype (56).

**Streptococcus bovis: **Streptococcus bovis (S.bovis) as a Gram-positive bacterium is about 10% of microflora in gastrointestinal track in adults and can induce disease. This bacterium caused-endocarditis and may lead to colorectal cancer in humans. As well, the presence of serum antibodies S.bovis was seen in patients with colorectal cancer, though the DNA of bacteria was not detected using PCR (57). It seems that the chronic inflammation process through the production of inflammatory cytokines in the colonic mucosa is the important factor of cancer (58). The studies have shown that the frequency of non-neoplastic and neoplastic polyps in infected patients with S.bovis was 35% and 26% and other bacteria was 7% and 2%, respectively (57, 59). 

**Other bacteria: **Several studies have been conducted reporting some other bacteria considered as cancer risk factor such as Salmonella enterica serovar typhimurium (S. typhimurium) and gallbladder cancer (GBC). GBC affects women 2-4 times more frequency than men (60). Bacillus tropheryma whipplei associate with lymphoma and Borrelia vincentii associate with squamous cell carcinoma (SCC) (61, 62). Furthermore, Chlamydia trachomatis *(C. trachomatis)* can cause infections of female reproductive system of women. It is one of the common of the complications sexually transmitted pathogens in females. Some studies have found that infected patients with *C. trachomatis* have a higher risk of cervical cancer (63).

**Prevention Solution: **Multiple tools to fight infections, such as vaccination, safe injection practices, screening of blood transfusion, safe sex practices, antimicrobial treatments can have beneficial effects on the future burden of cancer related-infections which is definitely should be focused in future research.

 In conclusion, presently a large number of infectious agents have been identified which either cause or contribute to specific human cancers. Therefore, understanding of these factors and their interaction with cancer is pressing issue that should be dealt with. In this way, screening of infections in cancer tissues may open up areas of research to a better understanding of optimizing cancer control strategies. Since viral infections tend to be more chronic than bacterial infections, thus viruses have a more prominent role in the development of cancer.
